# Neotropical palms: from their conservation to economic potential

**DOI:** 10.3389/fpls.2024.1487297

**Published:** 2024-11-22

**Authors:** Kauanne Karolline Moreno Martins, Suelen Alves Vianna, Ana Flávia Francisconi, Matheus Scaketti, Enéas Ricardo Konzen, Maria Imaculada Zucchi

**Affiliations:** ^1^ Biology Institute, State University of Campinas – UNICAMP, Campinas, São Paulo, Brazil; ^2^ Genetics and Genomics Conservation Laboratory – UNICAMP/USP, Piracicaba, São Paulo, Brazil; ^3^ Renewable Acelen – Research & Innovation Department - Plant Genetic Breeding Sector, São Paulo, Brazil; ^4^ Department of Genetics, University of São Paulo – USP, Piracicaba, São Paulo, Brazil; ^5^ Center for Limnological, Coastal and Marine Studies, Interdisciplinary Department, Federal University of Rio Grande do Sul – UFRGS, Imbé, Rio Grande do Sul, Brazil; ^6^ Secretariat of Agriculture and Food Supply of São Paulo State, APTA, UPDR, Piracicaba, São Paulo, Brazil

**Keywords:** Arecaceae, genetic diversity, genomics, biotechnology, ecosystem services, breeding, genome, historical importance

## Abstract

Palms (Arecaceae) are an important group of plants widely distributed throughout the world. The Arecaceae family comprises a great diversity of species, however, many of them are threatened with extinction due to their unbridled exploitation in search of economically important resources. An overview of palms biology will be presented, with emphasis on genetics and genomic resources of several species, as well as their socioeconomic impact worldwide, highlighting the main advances in recent research. Our discussion also covers the demand for urgent measures toward conservation and preservation of palms since they play key roles in maintaining biodiversity and providing essential ecosystem services. Fundamentally, this article is to raise awareness about the importance of palms and to encourage the protection and conservation of these valuable species.

## Introduction

The Neotropical region is one of the six biogeographical regions of the planet, covering Central America, a large part of South America as well as the Caribbean islands, the Antilles and tropical areas of Mexico, presenting similarities in fauna and flora, and is considered an area with great biodiversity, having the largest number of species of both animals and plants (Lima et al., 2018). Among the great wealth of species we find palm trees (Arecaceae).

**Figure 1:**
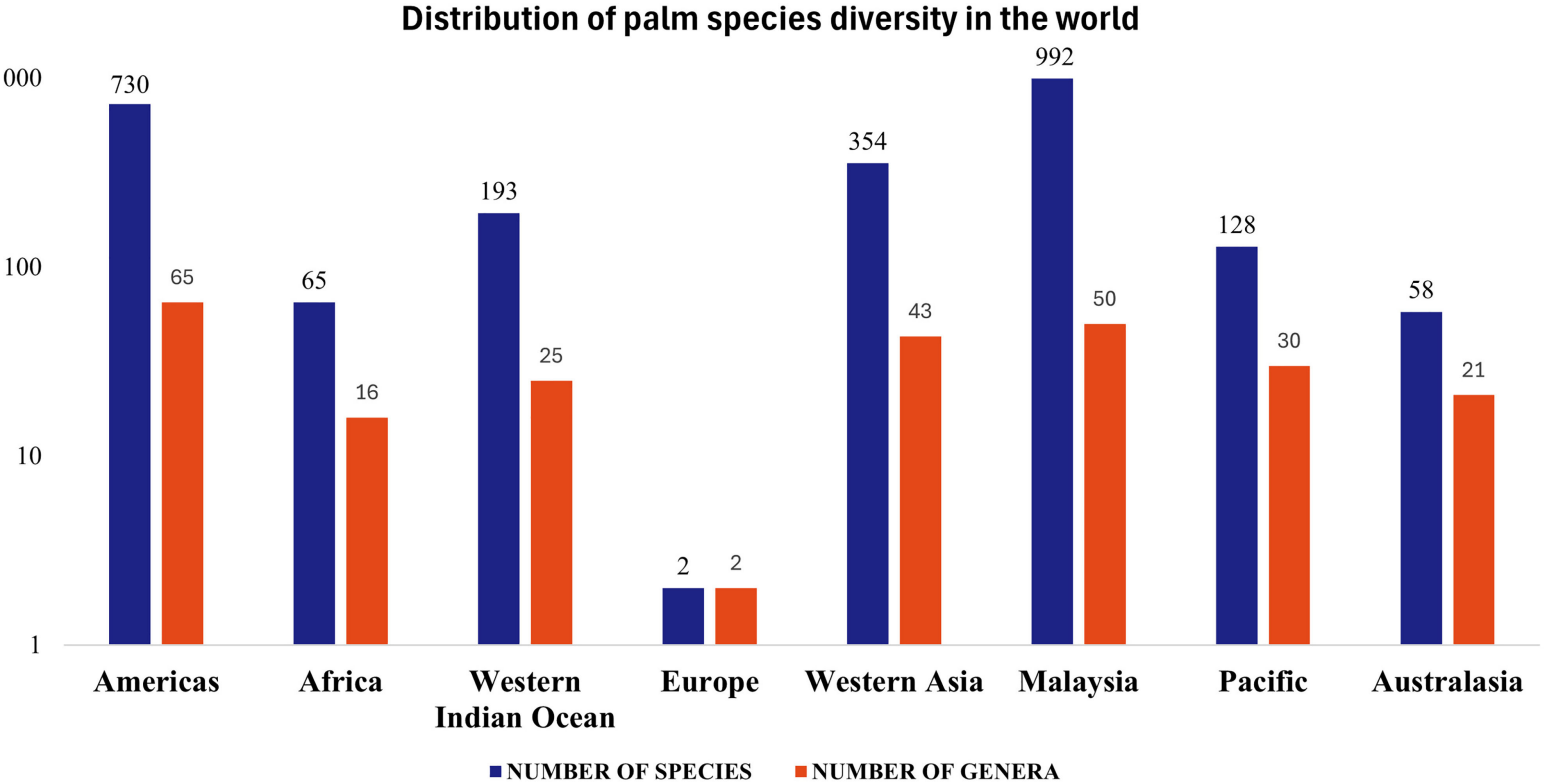
Distribution of palm species diversity in the world. Western Indian Ocean - Madagascar, Western Indian Ocean Islands; Continental Asia - Western Asia, Arabian Peninsula, Subcontinental India, Sri Lanka, Indochina, China; Malaysia - Malay Peninsula, Sumatra, Java, Borneo, Philippines, Sulawesi, Papua, New Guinea, Bismarck Archipelago, Solomon Islands; Pacific - New Caledonia, Fiji, Vanuatu, Hawaii; Australasia - Australia, New Zealand. The data is plotted on a logarithmic scale. Source: Dransfield et al. (2008).

The Arecaceae family, which comprises all palms, includes approximately 181 genera, with 2.600 species distributed in five subfamilies, Calamoideae, Nypoideae, Arecoideae, Coryphoideae and Ceroxyloideae (Baker and Dransfield, 2016). Among the wide variety of flora, the greatest diversity of genera and species of the family is distributed in regions with a tropical climate at low latitudes, influenced by environmental and biotic factors, and some taxa are found at higher latitudes, up to 44°N and S (Bjorholm et al., 2006; Henderson et al., 1995). Although it occurs predominantly in tropical and subtropical areas, the diversity of palm genera and species varies around the globe, with the greatest diversity of species (992) occurring in Malaysia followed by the Americas (730), with the latter surpassing the former in terms of the number of genera (65 versus 50) (**Figure 1**).

**Figure 2:**
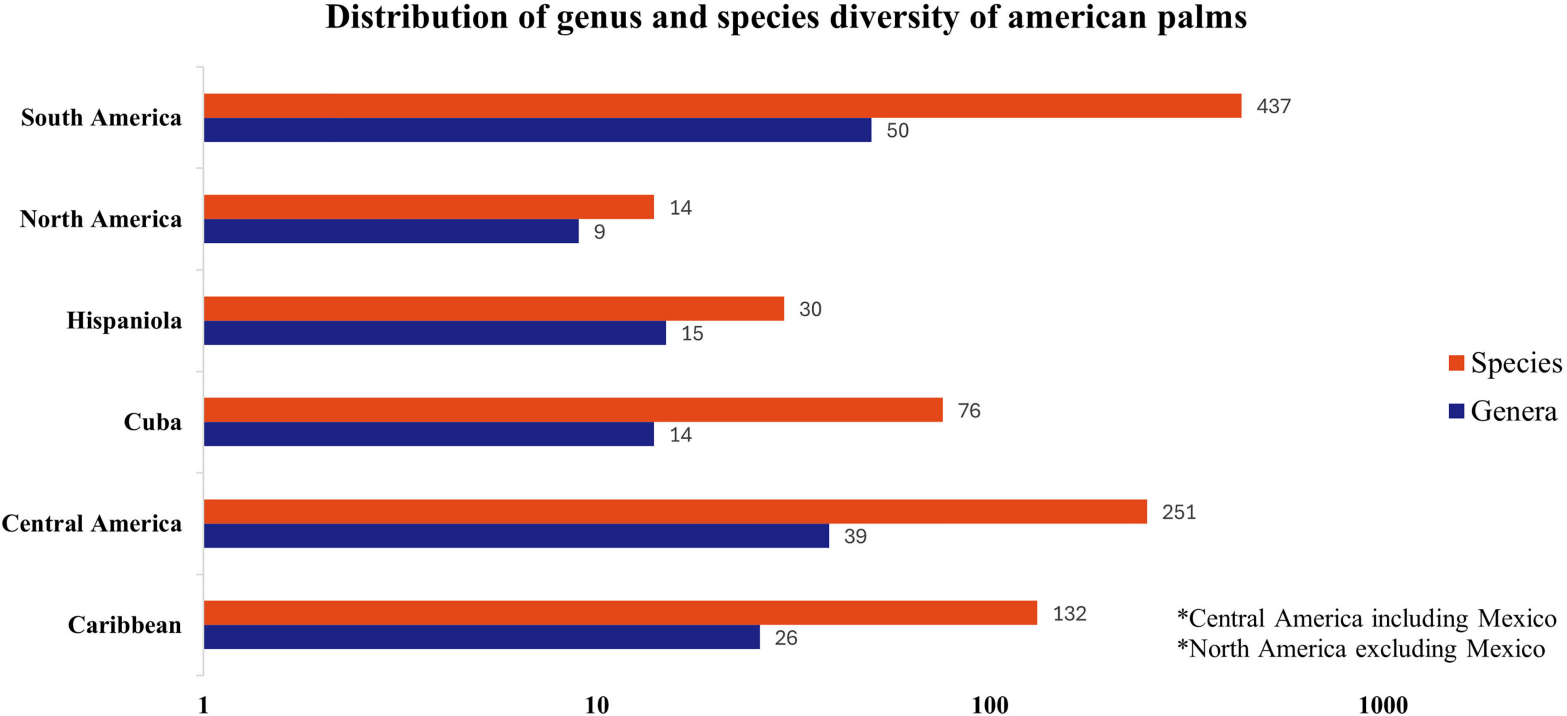
Distribution of palm species diversity in the Americas. The data is plotted on a logarithmic scale. Source: Dransfield et al. (2008).

Considering only the American continent, it can be seen that both the highest number of species and genera is recorded for South America (437 species - 50 genera), followed by Central America (251 species - 39 genera) (**Figure 2**).

Due to their wide distribution in tropical regions and diversity of forms, palm trees are of great importance to the functioning of ecosystems, participating in the structure and composition of vegetation and fundamental as a food resource for frugivorous animals and pollinators (Dransfield et al., 2008; Henderson et al., 1995; Zona and Henderson, 1989) and as a subsistence resource for various peoples in the form of food, wood and medicinal products (Uhl and Dransfield, 1987; Balick and Beck, 1990; Ellison, 2001). Several species are commercially exploited as non-timber forest resources providing different types of products such as wax, fiber, oil, ornamental and others, being cultivated on a large scale such as *Bactris gasipaes* for palm heart production (Galdino and Clemente, 2008) and others with regional use such as *Astrocaryum aculeatum* G.Mey. pulp in the Amazon region (Didonet and Ferraz, 2014).

This research consists of a literature review on the potential of palm trees as a non-timber forest product, in order to encourage their knowledge, preservation and appreciation of the goods and services they provide to ecosystems and human beings.

## Methodology and strategies

Data on native Neotropical palms was collected from available printed literature and online databases listed throughout the manuscript. The taxonomic characterization and geographical distribution of the species cited were checked in specialized literature (Henderson et al., 1995; Dransfield et al., 2008; Lorenzi et al., 2010) and online flora databases (Tropicos.org, Flora do Brasil). The use of the palms was classified as: (i) human food (HF); (ii) animal food (AF); (iii) medicinal use (MU), (iv) construction (C), (v) ornamental (O), (vi) handicrafts (H) and, additionally indicated if the use of the species occurs only as (i) extractivism for local use, (ii) extractivism for commercial use. The main native, non-domesticated species with occurrence in two or more countries in the Americas (non-endemic) and with known potential use were considered for the article.

**Figure 3:**
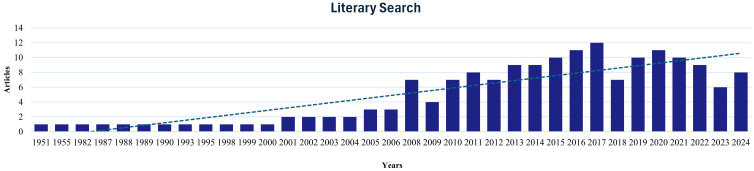
Graphic methodology. Number of articles per year.

Overall, this review was produced following four main steps: (i) identification of the proposed theme and problem question; (ii) establishment of criteria for analysis; (iii) selection of articles; and (iv) data interpretation and results. The basic problem questions of our review were: “What is the socioeconomic importance of palm trees? How can population genetic and genomic studies help us to understand the importance of these species?”. The selection of articles was conducted in Portuguese, Spanish and English using indexed platforms: National Library of Medicine (PubMed), Scopus, Scientific Electronic Library Online (SciELO) and Google Scholar. All articles were selected within a period of 40 years. Studies that did not contain the proposed theme, were incomplete or duplicated were excluded from this review. After the literary search, 162 articles were selected for the preparation of this review (**Figure 3**). The evaluation and analysis of the selected studies allowed the identification of variables, observations and data that gathered knowledge on the socioeconomic potential of palms. All data extracted from these articles were developed in a descriptive manner, observing, classifying, and organizing the knowledge generated on the properties of palms.

## Genera and species

**Figure 4:**
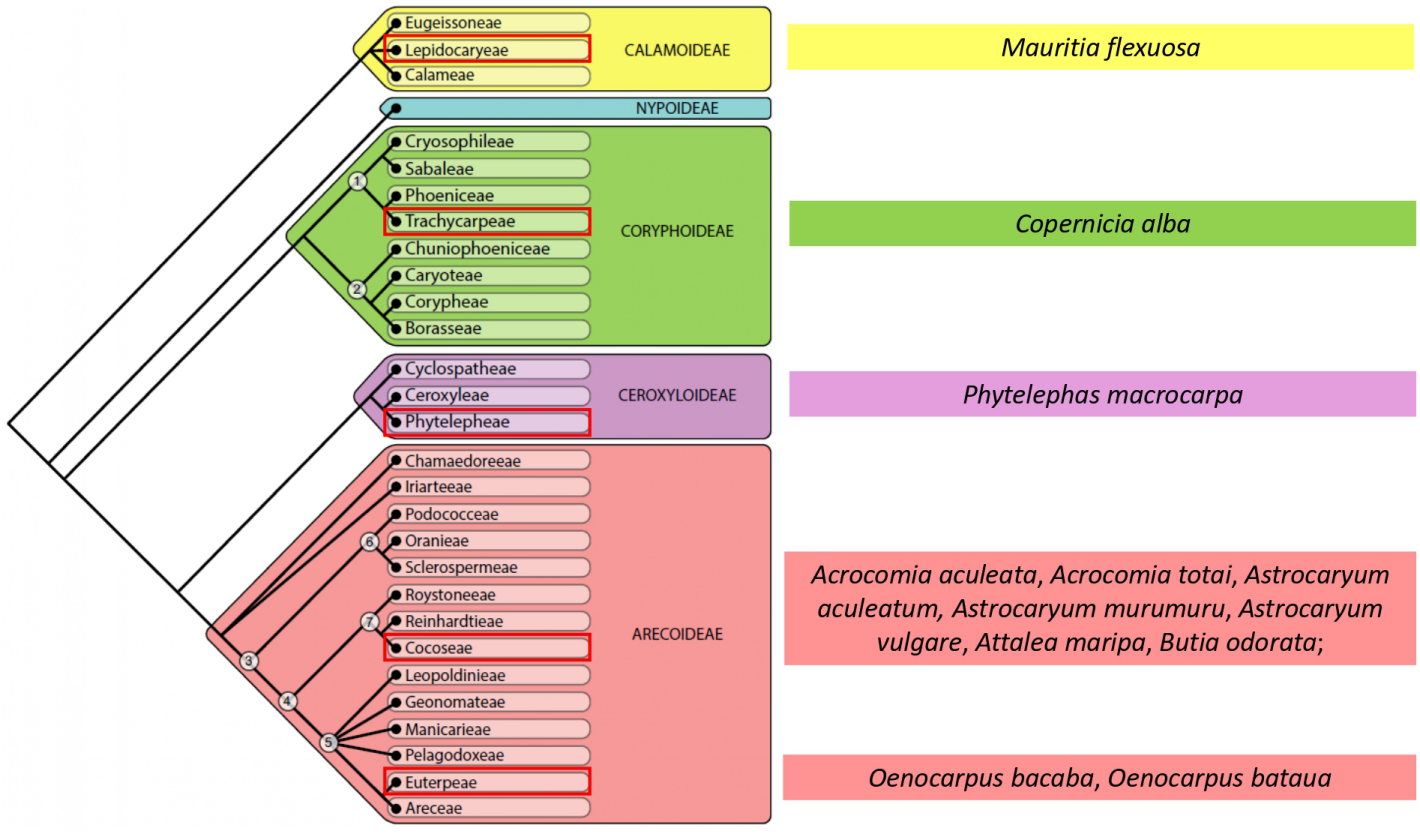
Arecaceae phylogeny at subfamily level with indication of species studied. Adapted from Baker and Dransfield (2016).

For this review, we considered 12 species distributed in 08 genera, occurring on the American continent and with a high potential for use. Phylogenetically, we have representatives from 4 of the 5 subfamilies that exist in Arecaceae, as shown in **Figure 4**.

## Taxonomy, morphological characterization and geographical distribution

### *Acrocomia aculeata* (Jacq.) Lodd. ex Mart.

The genus belongs to the subfamily Arecoideae, Tribe Cocoseae and Subtribe Bactridinae, the latter being represented by genera of spinescent palms (*Acrocomia, Aiphanes, Astrocaryum, Bactris* and *Desmoncus*) with a distribution restricted to the Americas, particularly South America (Dransfield et al., 2008). The species occurs in tropical and subtropical regions of the American continent, from Mexico and the Antilles to most of Brazil, covering different environments (Henderson et al., 1995, Vianna and Campos-Rocha, 2024).

The species is evergreen, heliophytic, occurring in greater densities in open areas, associated with pasture areas, also occurring in semi-deciduous forests and in places with rocky outcrops (Vianna and Colombo, 2013). Morphologically, it is easily recognized by the presence of thorns on the stipe and leaf sheaths in greater density. The plants range from 4 to 15 m in height on average, with cylindrical, ringed, spinescent stems and the presence of leaf sheaths from fallen leaves. The crown is made up of 20 - 40 pinnate compound leaves 4 - 5 m long, alternate leaflets unevenly distributed along the rachis, which gives the leaves a feathery appearance, often with some thorns on both the rachis and the leaflets, and senescent leaves are often attached to the plant. Inflorescences are interfoliate and branched, with several rachises bearing female flowers at the base and male flowers at the apex of the rachises, making it a monoecious species. The fruits are drupe, globose, 3-5 cm in diameter, with a brittle epicarp, usually brownish or yellowish-green in color; a fibrous, mucilaginous, yellow or orange mesocarp; a bony, dark endocarp, strongly adhered to the mesocarp; seeds with a large amount of solid endosperm, attached to the endocarp, with up to four seeds per fruit (Henderson et al., 1995; Lorenzi et al., 2010, Vianna and Campos-Rocha, 2024).

### *Acrocomia totai* Mart.

Belonging to the same subfamily, tribe and subtribe as *A. aculeata*, the species occurs only on the South American continent, restricted to Cerrado and Pantanal areas in parts of Bolivia, Paraguay, Argentina and Brazil (Vianna and Campos-Rocha, 2024). The palm can reach up to 15 m in height, with a discreetly ringed stem and thorns mostly on young plants, devoid of the remnants of the leaf sheath. The leaves may have thorns, especially on the rachis, with clear abscission. The leaflets are irregularly distributed and inserted in different planes, sometimes forming clusters of 2 to 3 leaflets along the rachis. The inflorescences are interfoliate and branched with the female flowers arranged at the base of the rachis, always forming triads, and the male flowers in the upper two-thirds of the rachis (Lorenzi et al., 2010). The fruits are globose, measuring 2.5-3.5 cm in diameter, with a brittle epicarp of varying colors, most commonly brown and shades of yellow and orange, a fibrous, mucilaginous mesocarp, also of varying color, a bony, dark endocarp and seeds with a large amount of solid endosperm (Vianna et al., 2021).

### *Astrocaryum aculeatum* G. Mey.

Similarly to *Acrocomia*, the genus belongs to the same group of spiny palms in the Arecoideae subfamily, Cocoseae tribe and Bactridinae subtribe, with geographical occurrence recorded for countries in the Amazon region, such as Brazil, Colombia and Venezuela (Dransfield et al., 2008). The solitary-stemmed palm can reach up to 25 m in height, with internodes covered in long, black spines up to 25 cm long. The leaves are pinnate, ascending, with the sheath and petioles covered in spines, the leaflets are irregularly inserted into the rachis in groups of 2-5 leaflets in different planes. The branched inflorescences are interfoliate with the peduncular bract also covered in spines, bearing female flowers in the basal region of each of the numerous rachillas. The bunches are large bearing hundreds of drupe fruits, globose to ellipsoid in shape, smooth or brittle epicarp, variable in color, measuring 3 to 8 cm in length by 2.5 to 6.5 cm in diameter, the mesocarp is fleshy, fibrous to slightly fibrous, yellow to reddish oleaginous, hard and black endocarp with a rounded seed, some fruits without seed or even with two seeds (Vianna, 2024a; Oliveira et al., 2022a).

### *Astrocaryum murumuru* Mart.

The spinescent palm occurs throughout the Amazon ecoregion from Colombia, Ecuador, Peru, Bolivia, Guyana, French Guiana, Suriname, Venezuela to Brazil. The species is cespitose, up to 15 m high, with pinnate leaves, the rachis, sheath and petiole covered in flat, black spines up to 30 cm long; numerous pinnules regularly inserted in the same plane. Inflorescences are branched, erect, with peduncular bracts densely covered in brown bristles and black spines and rachises bearing only one female flower at the base. The fruits are inverted conical, laterally flattened, rostrate, 6-8.5 cm long by 3.8-4.4 cm in diameter with an epicarp covered in brownish indumentum and bristles, a yellow, fleshy mesocarp, a hard, black, conical endocarp; the endosperm is also conical, homogeneous and whitish (Vianna, 2024a; Bezerra and Damasceno, 2022).

### *Astrocaryum vulgare* Mart.

A species that occurs predominantly in the eastern Amazon, it is cespitose, reaching a height of 20 m with internodes covered in long, black spines up to 22 cm long. The leaves are pinnate, with a sheath, petiole and rachis covered in partially black spines, irregularly inserted pinnules in groups of 2-6 on different planes. Inflorescences and infructescences erect; peduncular bract densely covered in spines, numerous rachises bearing 2-5 female flowers on the basal portion, each flanked by two possibly sterile male flowers, forming triads. Fruits globose to ellipsoid, 3.5-5 cm long × 2.5-4 cm in diameter, rostrate; epicarp smooth, orange to red; mesocarp orange, fleshy, fibrous and solid sweet endocarp, black; endosperm homogeneous (Vianna, 2024a; Oliveira et al., 2003).

### *Attalea maripa* (Aubl.) Mart.

Belonging to the subfamily Arecoideae, tribe Cocoseae, subtribe Attaleinae (Baker and Dransfield, 2016), it has been recorded as occurring in countries in the northern region of South America, predominantly associated with the Amazon region, and in Brazil it has also been recorded in some areas of the Cerrado. It is a tall palm, which can reach 25 m in height, with a solitary, smooth-surfaced stipe, with pinnate leaves, leaving part of the petiole attached to the stem for a long time. The pinnules are irregularly distributed in the rachis and inserted in different planes, giving the leaves a crisp appearance. Inflorescences are branched, interfoliate, and may have exclusively male, predominantly male, androgynous or predominantly female flowers. The fruits are ellipsoid, measuring 3.9-6.0 cm in length by 1.8-3.5 cm in diameter with a thin brown epicarp, a fleshy, fibrous and oily mesocarp, beige to yellow in color; an ellipsoid and bony brown endocarp and, externally, brown endosperm and internally whitish and solid (Soares, 2024; Matos et al., 2017).

### *Butia odorata* (Barb.Rodr.) Noblick

Belonging to the subfamily Arecoideae, tribe Cocoseae and subtribe Attaleinae, the species is restricted to southern South America (southern Brazil and eastern Uruguay). They are monoecious palms, with solitary or cespitose stems, pinnate, arched, grayish-green leaves and pseudopeciole with smooth or toothed margins. The leaflets are arranged in an ascending “V” shape in the rachis, distributed in the same plane. The inflorescences are branched, bearing male flowers throughout the rachis, with a greater concentration from the middle to the apex and the female flowers from the middle to the base of the rachis, forming triads with two males. The fruit can be ellipsoid, globose, oblong or ovoid with a yellow, orange, reddish, greenish or brown epicarp, a fleshy mesocarp, a brown endocarp and an ovoid or triangular endosperm (Heiden and Sant’Anna-Santos, 2024; Rivas and Barbieri, 2017).

### *Copernicia alba* Morong

As a curiosity, the genus was named after the rounded shape of its crown in honor of the astronomer Nicolaus Copernicius, who developed the heliocentric theory of the Solar System (Vianna, 2024b). *Copernicia alba*, is a South American species with a distribution restricted to Bolivia, Argentina, Paraguay and a small part of Brazil, associated with places with higher humidity such as riparian and gallery forests, including the Pantanal wetlands, occurring in some areas in high population densities, some only with individuals of the species (monodominate formation) forming the so-called “carandazal” (Tropicos.org, 2024; Vianna, 2024b).

The palm has a columnar, grayish and solitary stem, covered entirely by the remnants of the base of the fallen leaves, with a very typical appearance. The crowns are characteristically spherical, with flabelliform (fan-shaped) leaves, waxy on the abaxial side, grayish-green in youth and green with long petioles and spines on the sides of the rachis. The inflorescences are interfoliate, branched, longer than the leaves, with rachis bearing hermaphrodite flowers. The fruits are berry-like, ovoid, small (1-2 cm long), with a smooth, black epicarp, a thin, grayish-white mesocarp and a light brown ovoid seed about 1.2 mm long (Vianna, 2024).

### *Mauritia flexuosa* L.f.

A species belonging to the subfamily Calamoideae, tribe Lepidocaryeae and subtribe Mauritiinae, very characteristic of landscapes with wetter or periodically flooded areas in South America (Henderson et al., 1995; Dransfield et al., 2008). It is a dioecious palm, 3-25 m tall, with a solitary, smooth stem and aerial roots at the base. Large flabelliform (fan-shaped) leaves, broken into 45-230 segments. The inflorescences are interfoliate, branched up to the second order level, with the branched part measuring 1.4 - 2.4 m. There are staminate inflorescences (male flowers) and pistillate inflorescences (female flowers) on different plants. The fruits are ellipsoid-oblong (oval), 3.5-5.5 cm in diameter, epicarp characteristically covered in overlapping scales, reddish-brown in color, thin, fleshy, yellow-orange mesocarp, undifferentiated endocarp and seed with solid, homogeneous endosperm (Vianna, 2024c; Rossi et al., 2014; Storti, 1993).

### *Oenocarpus bacaba* Mart.

A monoecious palm belonging to the subfamily Arecoideae, tribe Euterpeae with a distribution restricted to the Amazon region in South America, it is even considered one of the hyperdominant species in the Amazon (Tropicos.org, 2024; Steege et al., 2013). Solitary or cespitose species, 12-25 m tall, with an erect stem, densely covered with fibers resulting from the decomposition of the leaf sheaths when young. Pinnate leaves with pinnules regularly distributed in the same plane, or grouped and arranged in several planes, silver-gray on the underside. Inflorescences are infrafoliate when in bud and infrafoliate, branched at first order level when open, bearing triads of flowers (one central female and two lateral male) in their basal portion and male flowers arranged in pairs or solitary in the apical portion. The bunches are large, about 1.5 m long, with hundreds to thousands of ellipsoid or globose, dark purple fruits, with a whitish, oily mesocarp and kernels surrounded by a thin, fibrous endocarp (Henderson et al., 1995; Lorenzi, 2024).

### *Oenocarpus bataua* Mart.

A palm species native to the Amazon region and occurring in Central and South American countries (Henderson et al., 1995) with a solitary, smooth stem, 5-25 m high with fasciculated roots at the base and no palm heart at the top. The leaves are pinnate, greyish and waxy, distributed on the same plant, with a persistent sheath on young plants and fibers on the margins and long fibers with a woolly aspect on the ligule. The inflorescences are intrafoliolar, pendulous, branched, with part of the rachis bearing male flowers forming diads and the other with two male flowers flanking a female flower forming triads. The fruit is an oblong to ellipsoid drupe of variable size (2.5 - 4.7cm × 2.0 - 2.5cm), with a smooth epicarp, green or violet when ripe, covered in a thin, whitish, waxy layer; the mesocarp is fleshy, white, greenish or purplish in color and the seed varies from ovoid-ellipsoidal to globose, covered in flattened fibers (Oliveira et al., 2022b; Maciel et al., 2021; Lorenzi, 2024).

### *Phytelephas macrocarpa* Ruiz & Pav.

A species known as “vegetable ivory” due to the appearance of the seed, its name is also a reference to elephants (Phyto = plant and elephas = elephant). It is a small, dioecious palm, up to 5 m tall, with a solitary, thick stem, rarely cespitose or underground with apparent adventitious roots and the presence of the remnants of the base of fallen leaves. Leaves are pinnate, arranged in a single plane with a fibrous sheath. Dimorphic inflorescences: the male inflorescence is spiky, and the female inflorescence is branched, bearing intensely fragrant flowers. The bunches are large, formed by aggregate fruits covered with projections similar to large woody aculei, with a fleshy mesocarp and a yellow color. Each fruit contains 8-12 seeds about 2 cm in diameter, with liquid and clear endosperm when the seed is immature, becoming solid at maturity, with an opaque white color which gives it an ivory-like appearance (Vianna, 2024d, Costa et al., 2006).

## Historical and socioeconomic significance of palms

### Historical importance

The Arecaceae family is distributed throughout the world, mainly in tropical and subtropical areas, such as the equatorial coast of Africa, Oceania, the Brazilian coast, the Amazon, Indonesia, and the Antilles (Moore and Uhl, 1982). A previous report showed that in 2008 the main cultivated species are oil palm (*Elaeis*), coconut (*Cocos nucifera*), date palm *(Phoenix dactylifera*) and betel palm (*Areca catechu*) with accounted for 14,585,811, 11,208,072, 1,264,611 and 834,878 hectares respectively (Food and Agriculture Organization of the United Nations, 2010).

**Figure 5:**
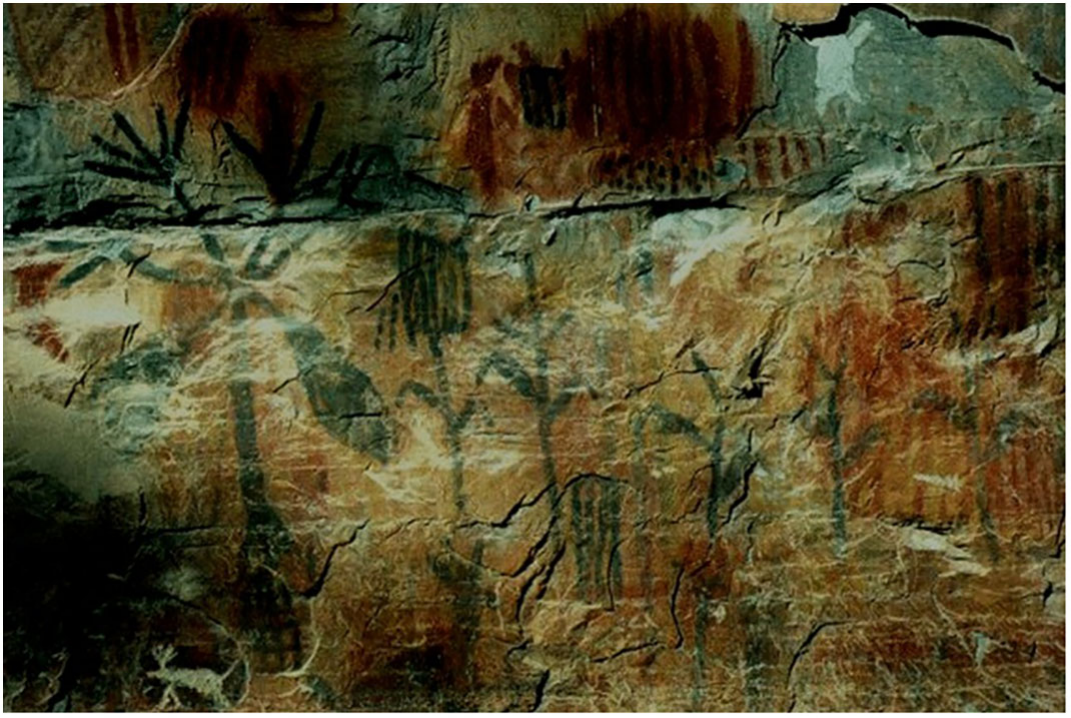
Archeological record. Rock paintings of palm tree *Mauritia flexuosa* (Buriti) from the Peruaçu Valley, Minas Gerais – Brazil. Worldwide records of plants are very rare, which makes the Peruaçu Valley a very particular place. Photo: Fábio O. Freitas/Embrapa Cenargen.

The use of palms by humans dates back to pre-historical times. Ever since, in addition to being used directly from nature, palms have been significantly important to the world, taking part in the daily lives and lifestyles of millions of people (Campos and Albuquerque, 2021). This is recorded in many ancient documents, just as shown in **Figure 5**, in which archaeologists found rock paintings representing the use and importance of palm trees for pre-historic populations.

### Traditional uses

The traditional medicinal uses that have been referred to most of palms here reviewed have influence research projects devoted to unraveling bioactive compounds of palms (Campos and Albuquerque, 2021). For centuries, in different communities around the world, they have been used due to their effectiveness against numerous diseases (Sen and Samanta, 2014). Since Ancient Mesopotamia and Egypt, and even in China, these plants have played a substantial role in medicine, as they have been constantly used in preventive medicine for the treatment and cure of acute and chronic diseases (Yang et al., 2018).

According to the World Health Organization (2019), the use of traditional medicine practices is increasingly growing. The major uses of palms for that purpose are through extracts and oils. That said, studies have initiated the isolation of molecules from bioactive compounds to understand and formulate new herbal medicines for treating infectious and chronic diseases (Dias et al., 2012). The pharmacological potential of palms is due to their secondary metabolism, that produces several compounds of pharmaceutical interest (Kabera et al., 2014). Moreover, some of them are biotechnologically exploited, to produce biodiesel, enzymes, among others, where there is the ultimately expansion of the use of plants by humanity (de Menezes et al., 2016).

*Butia* palms can be used in traditional medicine considering their antibacterial, anti-inflammatory and antioxidant activity (Schneider et al., 2017). The *Euterpe* genus comprises palms reported for their anti-inflammatory, anticancer, antimicrobial, antinociceptive, anticancer and anti-atherogenic, among others (de Almeida et al., 2020; Cardoso et al., 2018). *Elaeis* palm studied for antiparasitic and cicatrization abilities (Reddy et al., 2019). The *Syagrus* genus is widely used by communities as a nutritional source (Coimbra and Jorge, 2011). Moreover, *Syagrus* palms are also therapeutic (Hughes et al., 2013), being a good option for antiparasitic activities (Rodrigues et al., 2011), antioxidants (Belviso et al., 2013) and hypoglycemia (Lam et al., 2008). It is important to emphasize that although traditional communities use these species for different treatments, research should be conducted to prove their effectiveness, which requires much more work on the great diversity of molecules available from such plants.

### Biotechnological applications

With the use of palms by traditional communities as medicinal compounds, the pharmaceutical industries began to apply natural compounds such as nut oil and leaves/fruit extracts in their formulations. Through biotechnological tools, it is possible to improve the desirable characteristics of palms performing the isolation of bioactive molecules (David et al., 2015), e.g. Genetic Engineering: by modifying the genetic makeup of palm species, it’s possible to enhance the oil yield and modify the fatty acid composition to produce better-quality biodiesel. Techniques like CRISPR/Cas9 can be used for precise genome editing (Zhu et al., 2020). Omics technologies are also a viable alternative, such as: (i) Genomics: Sequencing the genomes of palm species to identify genes associated with oil production and quality (Schmutz et al., 2010); (ii) Transcriptomics: Studying gene expression profiles under different conditions to identify regulatory networks that control oil biosynthesis (Wang et al., 2009); (iii) Proteomics: Analyzing the protein profiles to understand the enzymes involved in oil production (Lucas et al., 2015); (iv) Metabolomics: Profiling metabolites to identify key intermediates and end-products in oil biosynthesis pathways (Fiehn, 2002).

Tissue culture and genetic transformation have been employed to produce plants with desirable traits such as improved resistance to pests, drought and diseases, higher productivity and quality of fruits and seeds (Patnaik et al., 1999; Gulzar et al., 2020). An important case of study is the African oil palm, which proved to be an important species to be invested for plant breeding, tissue culture, machinery, agrochemicals, oil fractionation and oleochemistry (Corley and Tinker, 2003). This oil palm presents the highest yielding oilseed crop, averaging 3-4 tons per hectare/year of mesocarp oil. This means that its productivity is higher than most oilseeds (Wahid et al., 2005).

*Syagrus* also holds great potential. These palms produce several bioactive compounds, properties capable of assisting in the treatment of bacterial and parasitic infections (*Syagrus coronata*); and are effective in the treatments and/or prevention of diseases such as diabetes and Alzheimer’s (*Syagrus romanzoffiana*), as it presents anticholinesterase and anti α-glycosidase activity (Coriolano et al., 2021).

### Other applications and what is needed

In general, due to their variable chemical composition, biotechnological tools are much promising for the production of considering the production of high-quality biodiesel (de Menezes et al., 2016), e.g. (i) Enzyme Engineering: Engineering enzymes involved in lipid metabolism to enhance their activity or specificity, thereby improving the efficiency of oil extraction and conversion to biodiesel (Bornscheuer et al., 2012); and (ii) Lipidomics: This is a subset of metabolomics focused specifically on lipids. It helps in understanding the detailed lipid composition and how it can be optimized for biodiesel (Han and Gross, 2005). However, it is interesting to note their importance for other areas, as pharmaceutics, for producing low-cost and more efficient drugs and components. However, to achieve such a goal, much more research is necessary to better acknowledge and understand the genetic diversity and structure of natural populations of several palms. That research is fundamental for next endeavors toward genetic improvement for improving cultivation and productivity of desirable products and molecules. Several potential products are listed in **Table 1**.

**Table 1:** Possible applications of American palm species.

Specie	Plant part utilized	Possible application	References
*Acrocomia aculeata*	Stipe	Construction, production of fermented drink (sap)	Brasil (2015); Coutiño et al. (2020)
	Mesocarp (pulp)	Human food (fresh or processed), Animal feed (cake) oil, biofuel, medicinal	Sant’Ana et al. (2023); César et al. (2015); Dias et al. (2021)
	Endocarp	Activated carbon, bioenergy, crushed stone	Vieira et al. (2021); Alves et al. (2022); Calvani et al. (2020)
*Acrocomia totai*	Thorns	Medicinal potential	Souza et al. (2017)
	Leaves	Bioenergy, medicinal potential	Souza et al. (2021); Souza et al. (2019)
	Mesocarp (pulp)	Human food, oil, biofuel, animal feed	Lapuerta et al. (2014); Peralta et al. (2013); Markley (1955)
*Attalea maripa*	Stipe	Bioenergy	Nagaishi et al. (2019)
	Apical meristem (palm heart)	Human food, animal feed	Lucas et al. (2023); Schons et al. (2024)
	Mesocarp (pulp)	Human food (fresh or processed), animal feed, oil	Lucas et al. (2023); Barbi et al. (2019)
*Astrocaryum aculeatum*	Epicarp (shell)	Catalyzer (biodiesel production)	Mendonça et al. (2019)
	Mesocarp (pulp)	Food (fresh or processed), animal feed, drink, oil, insecticide, medicinal	Brasil (2015); Santos et al. (2015); Santos et al. (2018); Linhares et al. (2017); Carneiro et al. (2017); Guex et al. (2020)
	Endocarp	Bio-jewelry, activated carbon	Brasil (2015); Santos et al. (2018); Umpierres et al. (2017)
	Endosperm (kernel)	Oil (cosmetic), medicinal, biofuel	Santos et al. (2018); Freitas et al. (2022)
*Astrocaryum murumuru*	Leaves	Cellulose industry	Bezerra (2012)
	Endosperm (kernel)	Oil, cosmetics, animal feed	Santos et al. (2023); Bezerra (2012); Serra-Ferreira et al. (2020);
*Astrocaryum vulgare*	Stipe	Civil construction	Lima et al. (2013)
	Endocarp	Bio-jewelry	Lima et al. (2013)
*Butia odorata*	Mesocarp (pulp)	Human food (fresh or processed), medicinal, antibiotic	Barbieri et al. (2014); Maia et al. (2019); Silveira et al. (2022); Wagner et al. (2022)
*Copernicia alba*	Leaves	Handicrafts (fibers)	Bortolotto et al. (2019)
	Endosperm (kernel)	Animal feed, oil, xylitol	Gorostegui et al. (2011); Castellani et al. (2014); Silva et al. (2023)
*Mauritia flexuosa*	Mesocarp (pulp)	Human food (fresh or processed), animal feed, oil, biofuel	Brasil (2015); Freitas et al. (2020); Morais et al. (2019); Costa et al. (2021); Lima et al. (2017); Amarante and Braga (2021); Santos et al. (2015)
	Endosperm (kernel)	Oil, insecticides	Santos et al. (2015)
*Oenocarpus bacaba*	Stipe	Construction, tools, medicinal	Miller (2002); Vasconcelos et al. (2015); Mans et al. (2022)
	root	Medicine	Vasconcelos et al. (2015); Leba et al. (2016)
	Epicarp (shell)	Drink	Baldissera et al. (2023)
*Oenocarpus bataua*	Inflorescence/infructescence rachis	Animal feed (salt supply)	Oliveira et al. (2022b); Miranda et al. (2009)
	Mesocarp (pulp)	Human food, functional food, oil, insecticide	Oliveira et al. (2022b); Rezaire et al. (2014); Montúfar et al. (2010); Santos et al. (2015)
	Endosperm (kernel)	Human food, oil, handicrafts, bio-jewelry	Oliveira et al. (2022b); Montúfar et al. (2010); Miranda et al. (2009)
*Phytelephas macrocarpa*	Leaves	Construction (roof), ropes (fibers)	Ferreira (2005)
	Mesocarp (pulp)	Human food, animal feed	Ferreira (2005)

## Molecular tools in genomics and molecular breeding

### Population genomics

Advances in molecular and genetic research have provided a better understanding of the genetic diversity and evolution of plant species, as well as their potential use in various areas. DNA molecular analysis has been used to investigate genetic diversity in palms. Currently, population genetic and molecular breeding studies have mainly been based on with next-generation sequencing techniques (Azizi et al., 2020). Such studies have allowed the identification of important genes for resistance to pests and diseases (Murphy, 2014). To set an example, the ongoing research on genetic diversity and adaptation in oil palms (*Elaeis guineensis* and *Elaeis oleifera*), that are important sources of vegetable oil, have delivered numerous findings in the literature. The genome sequencing studies of these species have identified genes responsible for oil production (Dussert et al., 2013).

Palms are an important source of genetic variability and can be used to improve existing palm species and develop new varieties. Germplasm banks have been established to preserve and document palm genetic diversity worldwide. Preservation and documentation of palm genetic resources are critical to ensure sustainable use of plant genetic resources (Jaradat, 2015).

Palm population genomics has become an area of great interest for researchers in recent years, since palms present a wide genetic and morphological diversity, besides having great economic importance in several regions of the world. Thus, population genomics can be used to understand the population structure, genetic diversity, and ecological adaptations of palms.

According to Cutter (2013), population genomics can be used to explore phylogenetic and biogeographic relationships, as well as to investigate genetic variation among populations and identify the molecular adaptations that allow species to adapt to different environments. In addition, population genomics can help to understand how climate change may affect palm distribution and survival, thus enabling the implementation of more effective conservation measures.

Baker et al. (2011) reported the use of population genomics in palms to elucidate genetic diversity in different species, including the identification of genetic polymorphisms and the analysis of gene flow between populations. They also highlight the importance of population genomics in the conservation of endangered palm species, enabling the monitoring of genetic variation and decision-making on management actions.

### Breeding

Since the early 1900s, plant breeding has played a fundamental role in the scientific and social field (Tester and Langridge, 2010). In agriculture, radical climate changes such as heat and drought have been directly affecting farmers worldwide, causing a decline in their productivity and yield (Swaminathan and Kesavan, 2012). Therefore, through molecular advances, plant breeding becomes a resource capable of developing plants with characteristics to adapt and develop under different environmental conditions (Bharadwaj, 2016), that is, a breeding toward climatic resilience.

Plant selection has been used in society for more than 10,000 years to create and identify plants with better nutritional values, a method aimed at improving the status of different plants (Moose and Mumm, 2008). Through scientific developments, it is possible to obtain different innovations in plant breeding (Varshney et al., 2006), making future foods and products to be increasingly based on natural plants (Li et al., 2018), such as with palms (E.g. *Bactris gasipaes*, *Euterpe edulis*, *Acrocomia aculeata*).

Finally, population genomics can also be applied in genetic improvement studies in palm trees, as reported by Yue et al. (2021). Population genomics can be used to identify traits of economic interest, such as resistance to diseases and tolerance to environmental stresses, thus allowing the selection of individuals with desirable traits for commercial cultivation. Basically, population genomics has a great potential in understanding the diversity, evolution, and conservation of palm trees, besides being able to be used in breeding programs for the commercial cultivation of these species. Currently, there are several programs for genetic improvement in palm trees, aiming to select individuals with desirable characteristics, such as resistance to diseases, higher fruit and seed production, greater resistance to environmental stress, among others.

Palm tree breeding programs are important tools to increase the productivity and resistance of the species, thus guaranteeing their conservation and the sustainability of the ecosystems that shelter them. The domestication of species and genetic improvement studies become attractive for financial investments in research and biotechnology. In this way, studies that opt to work with the diversity and genetic structure of natural populations can help in the development of strategies that prioritize the preservation and conservation of these important species.

Genetic improvement of palm trees has seen significant advancements through various scientific studies, aiming to enhance traits such as disease resistance, yield, and stress tolerance. One notable example is the work on oil palm (*Elaeis guineensis*) in Malaysia, where researchers have utilized marker-assisted selection to identify and propagate individuals with desirable traits. Studies by Rajanaidu et al. (2000) demonstrated the use of molecular markers to accelerate breeding programs, leading to the development of higher-yielding and more disease-resistant oil palm varieties. Another case involves the peach palm (*Bactris gasipaes*) in Brazil, where genetic improvement efforts have focused on increasing fruit size and quality (Gazel-Filho and Lima, 2001). Research by Clement et al. (1988) employed genetic diversity studies to select superior genotypes for cultivation, resulting in improved varieties that support local economies and food security.

Furthermore, genomic studies on date palms (*Phoenix dactylifera*) in the Middle East have identified key genes associated with drought tolerance and fruit development. Al-Dous et al. (2011) sequenced the date palm genome, providing a valuable resource for breeding programs aimed at enhancing resilience to climate change. These case studies underscore the potential of genetic improvement in palms, leveraging advanced genomic tools and traditional breeding techniques to meet agricultural and economic demands.

### Plastidial genomes

The plastome is represented as a single circular molecule composed of two inverted and repeated regions (IR), a large single copy (LSC) and a small single copy (SSC), with 120 to 130 genes, encoding ribosomal RNA (rRNA), transfer RNA (tRNA) and peptides. The size of the plastome can reach from 107 kb to 218 kb, varying between species (Menezes et al., 2018). According to Barrett et al. (2016), Arecaceae plastomes have a low rate of variation, however, the rate of diversification in palm genera seems to increase and a convergent evolution has been reported among species over the years (Ma et al., 2015; Faurby et al., 2016). Although most plastids are conserved, some events have been described, such as new RNA addition, loss of introns and high divergence of genes (He et al., 2016; Chen et al., 2017). This factor may be related to adaptive changes in plastid genes; however, it is still unknown whether the adaptations in the genus are related to environmental conditions (Lopes et al., 2018). Studies show an increasing number of new species with plastidial genomes (plastomes) becoming available, a useful tool for phylogenetics and evolution studies, as it assesses gene content, arrangements in genomes, gene loss and transfer, and recombination events (Park et al., 2017; Lopes et al., 2017). Moreover, hypervariable regions in plastomes can provide information that elucidates unclassified phylogenetic relationships (Smidt et al., 2020). The complete sequence of plastomes is a prerequisite for the selection of target intergenic regions for transgene insertion, moreover, it has been used in metabolic engineering (Daniell et al., 2016) and presents itself in a feasible way for the manipulation of plastidial biosynthesis and fatty acids (Rogalski and Carrer, 2011). Therefore, knowledge on plastomes has important applications in biotechnology (Daniell et al., 2016; Zhang et al., 2017). Plastome assembly becomes effective for understanding evolutionary aspects of plants at the gene level (Park et al., 2017).

### Genome assembly

Genome assembly is a method that has been increasingly used in plant breeding and population genomics. It allows the reconstruction of an organism’s DNA sequence, which can lead to important discoveries regarding genome structure and function (Zhang et al., 2016). Genome assembly has been proven a valuable tool in identifying genes and genetic variants associated with desirable traits, as well as understanding the genetic structure of plant varieties (Elshire et al., 2011). As for traits, genomes resequencing has assisted in the discovery of genomics regions associated with disease resistance and tolerance to environmental stresses (Song et al., 2023). This allows the creation of plant varieties that are more resistant and adapted to specific environmental conditions.

It is well established that population genetics is a field of genetics that deals with changes in allele frequency in populations over time. In turn genome assembly adds up as an important resource to the field as it allows a deeper understanding of the genetic structure of populations and the identification of molecular markers associated with specific traits (Allendorf et al., 2010; Wright, 1951).

As for palms, an example of the application of genome assembly in population genetics is the study by Al-Mssallem et al. (2013), that investigated the genetic diversity in *Phoenix dactylifera* L. The results of this study revealed a great genetic diversity in these populations, as well as the presence of adaptive genes associated with traits such as disease resistance and tolerance to environmental stresses.

It is valid to emphasize the importance of genome assembly as a tool supportive of conservation of the biodiversity of palm species, since it allows a thorough characterization of the genomic diversity status and the genomic structure of populations under investigation, enabling conservationists to design more effective and targeted conservation strategies (Dransfield et al., 2008).

Currently, there are some palm species with their genomes assembled and deposited to the National Center for Biotechnology Information (NCBI), 2023. Our search showed the following species with deposited assemblages: *Cocos nucifera*, *Elaeis guineensis*, *Elaeis oleifera*, *Phoenix dactylifera*, *Areca catechu*, *Phoenix roebelenii*, *Metroxylon sagu*, *Calamus simplicifolius*. It is important to note that the list may be constantly being updated, as new species may have their genomes assembled and made available to the scientific community.

### Phylogeny

Phylogeny is the study of evolutionary relationships among species and taxonomic groups, based on morphological, molecular and/or behavioral characteristics. In palms, phylogeny has been widely studied through molecular analyses, which allow the reconstruction of evolutionary history and the understanding of relationships between species (Baker et al., 2011). As palms are a diverse group of plants, distributed worldwide, that are important to humans as a source of food, raw material, and ornamentation, as well as playing a crucial role in many ecosystems, especially in tropical regions, phylogenetic studies prove to be extremely important for addressing several biological and genetic questions (Baker and Couvreur, 2012; Muscarella et al., 2019; Dransfield et al., 2008).

Phylogenetic analyses are key to the understanding of biological diversity. Additionally, it can also be used to identify species at risk of extinction and to plan more effective conservation strategies. This is because the most endangered species usually have older and/or unique evolutionary lineages, which makes them more important from a biological diversity perspective (Baker and Dransfield, 2016; Muscarella et al., 2019, Baker et al., 2009).

There are several methods for phylogeny reconstruction in palms, including DNA and RNA sequences, morphological traits and both combined (Baker et al., 2011; Muscarella et al., 2019; Eiserhardt et al., 2011). Molecular analyses are the most common and include:

- *Gene sequencing:* genes that are commonly used in palm phylogeny include *rbcL* (from chloroplast), *ITS* (internal transcribed spacer of nuclear DNA), *trnL-F* (from chloroplast) and *matK* (plastidial marker). These genes show different rates of evolution and allow comparisons among species at different taxonomic levels (Eiserhardt et al., 2011; Meerow et al., 2009).
- *Molecular markers:* in addition to genes, molecular markers such as microsatellites and SNPs (single nucleotide polymorphisms) are also used for phylogeny reconstruction in palms. The use of genetic markers together with DNA sequences has proven to be an efficient strategy for the inference of phylogenetic relationships in palm trees, allowing the resolution of evolutionary relationships at deeper levels and the identification of possible hybridization events (Muscarella et al., 2020).

According to Muscarella et al. (2020), phylogenetic inferences in palms have been used to understand the evolution of diversity and adaptations of these plants to different environments. Crucial information for understanding the evolution of palms and their taxonomic classifications.

In addition, comparative genomics among palm species has been used to identify common and unique features in their genomes, which can lead to a better understanding of palm evolution and better use of their genetic resources. Genetic analysis has also been used to differentiate species and subspecies, as well as to understand phylogenetic relationships among palm species (Dussert et al., 2013).

### Socio-economic impact

Palm trees are a group of plants with great socioeconomic importance worldwide, especially for populations living in tropical and subtropical areas. The Arecaceae family stands out for their diversity of uses, ranging from food production to the manufacture of cosmetic products and medicines.

Palm trees have been used by traditional communities for centuries, providing food, shelter, medicines, and other important resources. According to Eiserhardt et al. (2011), Arecaceae palms are important sources of edible oils, such as palm oil and coconut oil, which are widely used in cooking around the world. In addition, fibers from palm leaves are used in the manufacture of baskets, mats and other craft items and are also used in the manufacture of cosmetic products and medicines, such as palm kernel oil, which is used in the production of soaps and skin creams.

The direct socioeconomic impact provided by palm trees is the production of oil palm is an important economic sector in many tropical countries, contributing significantly to employment and income generation. According to Baker and Couvreur (2012), palm oil production is a major source of income for rural communities in many countries, including Indonesia and Malaysia.

In addition, oil palm production plays an important role in food security by providing an important nutritional supplement for low-income communities. According to Muscarella et al. (2020), oil palm production has been associated with significant improvements in the health and well-being of local populations. However, oil palm production is also often associated with negative environmental impacts, such as deforestation and degradation of natural habitat. It is important to implement sustainable oil palm production practices to minimize these impacts and ensure the conservation of natural areas.

## Economic, social, and environmental indicators of neotropical palms

### Economic indicator

- *Income and Livelihoods:* The economic impact of palm-derived products is substantial, particularly in rural economies. Direct income is generated from the sale of fruits, wood, fibers, and oils, while indirect income comes from associated activities such as tourism, crafts, and services. For example, the commercialization of açai (*Euterpe oleracea*) has provided significant revenue to Amazonian communities, transforming local economies and improving livelihoods (Brondizio, 2008). Similarly, the harvesting of palm hearts has become a vital economic activity in various neotropical regions.

- *Contribution to GDP:* Palm-related activities contribute markedly to local and national GDPs. This includes both primary production and secondary industries such as processing and marketing. For instance, in some regions of Brazil and Colombia, palm oil production is a major economic driver, contributing significantly to the agricultural GDP (Koh and Wilcove, 2008).

- *Employment:* The palm industry generates numerous jobs, both direct (cultivation, harvesting, processing) and indirect (marketing, distribution, services). It is essential to consider the variety of employment created, which includes temporary, permanent, skilled, and unskilled positions (Feintrenie et al., 2010). The sector’s ability to provide diverse employment opportunities helps in stabilizing local economies and reducing poverty. E.g. (i) Direct Employment: The cultivation and processing of palm oil in Indonesia generate a considerable number of direct jobs. These include roles in plantation management, harvesting, and processing facilities. According to Feintrenie et al. (2010), the oil palm industry in Bungo district alone supports a large workforce, with plantations employing a mix of skilled and unskilled labor. The jobs range from manual labor positions, such as fruit collection and processing, to more specialized roles in plantation management and machinery operation. (ii) Indirect Employment: In addition to direct employment, the palm oil industry also creates numerous indirect job opportunities. These include roles in marketing, distribution, and services related to the palm oil supply chain. Feintrenie et al., highlight that local economies benefit significantly from the ancillary businesses that support the palm oil industry. For example, transportation services for moving palm oil products, retail operations selling palm-derived goods, and maintenance services for plantation equipment are all essential components of the employment landscape. Employment Characteristics: Feintrenie et al., emphasizes the diversity of employment types within the palm oil sector. Employment opportunities include temporary and permanent positions, catering to a wide range of skill levels. The sector provides critical income sources for local communities, contributing to poverty alleviation and economic stability. Furthermore, the study notes that the oil palm industry tends to offer more stable and higher-paying jobs compared to other agricultural sectors, which is a significant factor in its preference among local farmers and workers.

- *Gender and Equity Considerations:* An important aspect of employment in the palm oil sector is the involvement of women and efforts toward equitable distribution of benefits. According to Cramb and McCarthy (2016), women’s participation in the palm oil industry is crucial, especially in roles such as nursery management, harvesting, and processing. These opportunities provide women with independent income sources, contributing to their empowerment and improving household economic conditions.

- *Marketing and Investment:* The volume of palm product production, market prices, and profit margins are critical indicators of economic health. Additionally, significant investments in plantations, processing facilities, infrastructure, and technology reflect the sector’s growth and potential (Santika et al., 2019). These investments not only boost production capacity but also enhance the quality and sustainability of palm products (Donald, 2004).

### Social indicator

- *Food Security:* Palm products play a crucial role in the diet and nutrition of local communities, thereby enhancing food security. Palm fruits are rich in essential nutrients, contributing to the sustenance and nutritional needs of rural populations (Johnson, 1998). For instance, the high-fat content in palm fruits can be critical for energy intake in regions where food scarcity is an issue.

- *Well-being*: Economic benefits derived from palm cultivation have a direct impact on the well-being of local populations. Improvements in living conditions, such as access to clean water, sanitation, education, and healthcare, are often linked to the economic gains from palm-related activities (Sayer et al., 2012). These improvements contribute to overall community health and development (Schroth et al., 2004).

- *Poverty Reduction and Equity:* Equitable distribution of benefits and the empowerment of women and vulnerable groups are essential for sustainable development. The palm sector has the potential to reduce poverty by providing income-generating opportunities and promoting social equity (Chao, 2012).

### Environmental indicator

- *Sustainable Management Practices:* Adopting environmentally sustainable practices in palm plantations is crucial for long-term viability. Certification schemes and sustainable management plans, such as those promoted by the Roundtable on Sustainable Palm Oil (RSPO), help mitigate negative environmental impacts (Koh and Wilcove, 2008). Sustainable practices ensure the preservation of natural resources and reduce the ecological footprint of palm cultivation (Gaveau et al., 2016).

- *Biodiversity Impact:* Understanding the impact of palm exploitation on biodiversity is essential for conservation efforts. Palm ecosystems contribute significantly to biodiversity, offering habitat and food resources for various species, (e. g. *Euterpe edulis*) (Scariot, 2015). The role of palms in climate regulation, soil protection, and water provision underscores their environmental importance (Fitzherbert et al., 2008). Efforts to maintain and enhance biodiversity within palm plantations can lead to more resilient and sustainable ecosystems (Struebig et al., 2015).

## Conclusion

Arecaceae, the palm family, is of great socio-economic importance around the world, providing crucial resources for local communities and contributing significantly to the global economy. Palm trees play a key role in maintaining biodiversity and ecosystem services, but many species are threatened with extinction. It is therefore essential to ensure that palm genetic resources are conserved in a sustainable way, as this minimizes negative environmental impacts and protects natural areas. The socio-economic importance of palm trees is evident, covering economic, social and environmental indicators. The economic indicators highlight that the income generated by palm-derived products, such as fruit, wood, fibers and oils, is vital to local economies. Palm trees also contribute to the GDP of the producing regions, reflecting their economic importance. The sector generates numerous jobs, providing essential employment opportunities. Social indicators emphasize the contribution of palm products to food security and general well-being, linking the economic benefits of oil palm cultivation to improved living conditions and poverty reduction. Environmental indicators highlight the importance of sustainable management practices, biodiversity conservation and the ecosystem services provided by oil palm ecosystems. Population genetic and genomic studies are also fundamental to understanding the importance of palm species. They reveal patterns of genetic variation that inform conservation priorities and develop effective management strategies. These studies facilitate the identification of genes associated with important traits, such as disease resistance and stress tolerance, which can improve sustainable palm cultivation. By integrating genetic and genomic data with ecological and biogeographical information, researchers can create comprehensive conservation and management plans that ensure the long-term viability of palm species and their ecosystems. In addition, preserving traditional knowledge and strengthening social capital through community cooperation are key to increasing the resilience and sustainability of palm-related activities. These measures stimulate community involvement and ownership, promoting sustainable development.

## Data Availability

The datasets presented in this study can be found in online repositories. The names of the repository/repositories and accession number(s) can be found in the article/supplementary material.
